# Effects of ursolic acid on sub-lesional muscle pathology in a contusion model of spinal cord injury

**DOI:** 10.1371/journal.pone.0203042

**Published:** 2018-08-29

**Authors:** Gregory E. Bigford, Andrew J. Darr, Valerie C. Bracchi-Ricard, Han Gao, Mark S. Nash, John R. Bethea

**Affiliations:** 1 The Miami Project to Cure Paralysis, Miami, Florida, United States of America; 2 Department of Neurological Surgery, University of Miami Miller School of Medicine, Miami, Florida, United States of America; 3 Department of Health Sciences Education, University of Illinois College of Medicine at Peoria, Peoria, Illinois, United States of America; 4 Department of Biology, Drexel University, Philadelphia, Pennsylvania, United States of America; 5 Department of Rehabilitation Medicine, University of Miami Miller School of Medicine, Miami, Florida, United States of America; Garvan Institute of Medical Research, AUSTRALIA

## Abstract

Spinal Cord Injury (SCI) results in severe sub-lesional muscle atrophy and fiber type transformation from slow oxidative to fast glycolytic, both contributing to functional deficits and maladaptive metabolic profiles. Therapeutic countermeasures have had limited success and muscle-related pathology remains a clinical priority. mTOR signaling is known to play a critical role in skeletal muscle growth and metabolism, and signal integration of anabolic and catabolic pathways. Recent studies show that the natural compound ursolic acid (UA) enhances mTOR signaling intermediates, independently inhibiting atrophy and inducing hypertrophy. Here, we examine the effects of UA treatment on sub-lesional muscle mTOR signaling, catabolic genes, and functional deficits following severe SCI in mice. We observe that UA treatment significantly attenuates SCI induced decreases in activated forms of mTOR, and signaling intermediates PI3K, AKT, and S6K, and the upregulation of catabolic genes including FOXO1, MAFbx, MURF-1, and PSMD11. In addition, UA treatment improves SCI induced deficits in body and sub-lesional muscle mass, as well as functional outcomes related to muscle function, motor coordination, and strength. These findings provide evidence that UA treatment may be a potential therapeutic strategy to improve muscle-specific pathological consequences of SCI.

## Introduction

Traumatic spinal cord injury (SCI) incites numerous pathophysiological changes and persistent metabolic abnormalities [1–5] that contribute to long-term effects on body systems. [6, 7] Physical limitations related to movement, musculoskeletal activity and weight-bearing contribute to deleterious changes in body composition, typified by rapid and long-term declines in metabolically active muscle-mass [8–16] and bone [17–23], as well as stark increases in central adiposity [24–27] which contributes to the maladaptive metabolic profile in SCI. As a result, there is a prevalence of co-morbid risk factors for *cardiometabolic* disorders in chronic SCI including obesity [28], dyslipidemia [24–26, 29–37], glucose intolerance, and insulin resistance [5, 36], symptoms that are indicative of cardiovascular disease (CVD) and diabetes. Notably, CVD has emerged as the leading cause of mortality in chronic SCI. [38, 39]

Sub-lesional skeletal muscles undergo severe pathological consequences following human SCI. Muscle mass is diminished by as much as 48% as early as 6-weeks post-SCI [13], and atrophy ranges from 30–60% depending on muscle type, duration and completeness of injury. [22] These changes coincide with muscle fiber-type transformation from slow oxidative Type I to fast glycolytic Type II [8, 12, 40–44], diminishing skeletal muscle oxidative phenotype that is linked to insulin resistance and progression of metabolic syndrome.

In SCI, a reduction in anabolic hormones such as testosterone, insulin and insulin-like growth factor-1 (IGF-1) [45, 46] may contribute to reduced IGF-1 receptor activation of metabolically-linked signaling pathways in muscle. It is well-established that activation of phosphoinositide-3 kinase (PI3K)/Akt and downstream anabolic target mammalian target of rapamycin (mTOR) regulate, in part, skeletal muscle protein synthesis, cell growth and metabolism. [47–49] Additionally, mTOR-activation regulates the transcription factor fork-head box protein 01 (FOXO1), which is associated with catabolic genes involved with ubiquitin-proteasomal degradation in muscle. [50] mTOR is a complex signal integrator that regulates both anabolic and catabolic processes in the muscle, however these processes are not well defined in SCI.

Recently, interest in the naturally occurring lipophilic pentacyclic triterpenoid ursolic acid (UA) in medical research has grown significantly due to its pharmacological effects and low toxicity. [51] For example, studies have shown that UA enhances insulin- and IGF-mediated phosphorylation of AKT, S6K1 and FOXO1 in skeletal muscle. [52, 53] Importantly, it was reported that UA inhibits skeletal muscle atrophy associated with denervation and induces skeletal muscle hypertrophy in the absence of an atrophy stimulus [53], in addition to its documented ability to augment muscle strength [54] and stimulate mTORC1 signaling in skeletal muscle. [55] Although implicated in skeletal muscle metabolic and growth signaling, the effects of UA on muscle pathophysiology has not been explored in SCI. In this study, we investigated UA as an anabolic stimulator of metabolically-linked signaling pathways in muscle and explored phenotypic outcomes in sub-lesional muscle following traumatic SCI. Our primary objectives are to examine following experimental SCI i. intracellular mTOR signaling, ii. the expression of related catabolic genes, and iii. the effects on these outcomes with UA treatment. We hypothesize that SCI will i. reduce growth pathways via mTOR signaling, ii. increase the expression of catabolic genes, and iii. UA will attenuate these SCI-induced changes. In addition, we aim to examine the effect of SCI on physical and functional measures, and whether UA-induced improvement in muscle signaling translates to improvements in these outcome measures.

## Materials and methods

All animal protocols were approved by the University of Miami Institutional Animal Care and Use Committee (IACUC) and are in accordance with National Research Council guidelines for the care and use of laboratory animals. Animals were group (socially) housed in a temperature- and humidity- controlled rodent vivarium, maintained on a reverse 12-hour light/dark cycle, and given food and water *ad libitum*. No additional environmental enrichment was provided due to its reported effects on the nervous system, including neurogenesis and levels of inflammation, which may affect experimental outcomes. Animals were acclimated for seven days prior to study experiments, which included being handled daily to get used to human contact and minimize distress. To ensure minimal suffering prior to any surgical procedure, to demonstrate that the animal was completely under anesthesia, we examined hind limb reflex by pulling and straightening the hind limb to determine when the limb is flaccid. Secondly, we pinched the hind paw for the lack of pain response. The flaccidity of the limb and the absence of reflex automatic movements at the pinching of the tail indicated a deep state of anesthesia. Following surgical procedures, animals were administered buprenorphine (0.1 mg/kg) BID for 3-days post surgery and PRN thereafter. Animals were examined twice daily for post-stress health status by observation of activity level, respiratory rate, and general physical condition. Body weight was monitored every other day. Excessive weight loss (>20%) and decreased grooming behavior was considered as criteria for early exclusion from the study and/or euthanasia. Physical conditions such as moribund state, dehydration, and anorexia were also considered as criteria for early termination. Euthanasia was carried out by carbon dioxide inhalation according to the recommendations for euthanasia detailed in the 2007 Report of the American Veterinary Medical Association’s (AVMA) Panel on Euthanasia. Euthanasia was performed in a manner to avoid animal distress. The chamber was not pre-filled with gas before placing the animals inside, and the rate of CO2 flow into the chamber was slowly increased. Animals were in a deep state of sleep after one minute, then the CO2 flow rate was increased for another four minutes. After CO2 exposure, confirmation of termination was accomplished by cervical dislocation.

### Traumatic SCI and UA administration

Surgeries were performed at the Animal and Surgical Core Facility of the Miami Project to Cure Paralysis according to protocols approved by the IACUC of the University of Miami. Contusion injury was induced with the Infinite Horizon Impactor device adapted to the mouse. The infinite Horizon impactor device has been established in producing precise, graded contusion, with reproducible lesion volume and functional outcomes assessed using Basso, Beattie, Bresnahan (BBB) and Basso Mouse Scale (BMS) [56] open-field locomotor rating scales. [57] In brief, Adult female C57Bl/6 mice (12-weeks old, ~20g; Jackson Laboratories) were anesthetized with an intraperitoneal injection of ketamine (80–100 mg/kg) and xylazine (10 mg/kg). Complete anesthetization was determined by the lack of a stereotypical retraction of the hind-paw in response to a nociceptive stimulus. Mice were then subjected to a laminectomy at vertebrae T9 and the exposed spinal cord was injured at a predetermined impact force of 70 kdynes (severe injury). Sham-operated animals underwent all surgical procedures, including laminectomy, but their spinal cords were not injured. After surgery, animals were housed separately and treated with subcutaneous lactated Ringer’s solution to prevent dehydration. Manual bladder expression was performed twice daily. Prophylactic antibiotic gentamicin was administered daily for 7 days to prevent urinary tract infections. SCI animals were randomized to 3 groups: SCI, SCI/UA (TCI America) [200 mg/Kg UA dissolved in corn oil (CO) (Spectrum Chemical Mftg Corp); i.p.], and SCI/Vehicle (CO only). UA was administered using a previously reported [53] dose that resulted in positive and effective changes in mTOR signaling and catabolic gene expression. UA/CO treatments were administered once daily until time of sacrifice. Animal tissue was harvested 1-, 2-, or 4-weeks post SCI, snap-frozen in liquid nitrogen and stored at –80°C until the time of assay.

### Protein extraction and immunoblot analysis

Mice soleus muscle tissues were harvested and homogenized in a Dounce homogenizer with extraction/lysis buffer (20 mM Tris–HCl, pH: 7.5, 150 mM NaCl, 1% Triton X-100; 1 mM ethylenediaminetetraacetic acid, 1 mM ethylene glycol tetraacetic acid, 2.5 mM pyrophosphate, 1 mM β-glycerophosphate) containing protease and phosphatase inhibitor cocktails and then centrifuged at 15 000 x g for 2 minutes. Lysates were mixed with 2x Laemmli loading buffer. Equal amounts of protein were resolved on 10–20% gradient Tris-HCl pre-casted gels, to separate proteins with a wide range of molecular weights, transferred to polyvinylidene fluoride (PVDF) membranes and placed in blocking buffer (0.1% Tween-20, 0.4% I-block in PBS) overnight. Membranes were then incubated with primary antibodies followed by the appropriate HRP-conjugated secondary antibody (1:1000). Visualization of the signal was obtained with an enhanced chemiluminescence substrate using a Phototope- HRP detection kit (Cell Signaling Technology®). Quantification of bands corresponding to phosphor-PI3K, phosphor-AKT, phosphor-mTOR and phosphor-S6K was made using the ChemiDoc Touch™ Imaging System (BioRad), and normalized to β-Actin, PI3K^Total^, AKT^Total^, mTOR^Total^, or S6K^Total^, where appropriate.

### Total ribonucleic acid (RNA) isolation and quantitative RT-PCR

Total RNA was isolated from mouse soleus using TRIZOL Reagent (Invitrogen) according to manufacturer’s instructions. 5 ug of RNA were reverse transcribed using SuperScript III First Strand Synthesis System for RT-PCR (Invitrogen). Real-time PCR was performed with the Applied Biosystems 7300 Real-time PCR System on samples amplified with Rotor-Gene SYBR Green PCR Kit (Qiagen) and primers for the transcription factor FoxO1, the muscle-restricted E3 ligases Atrogin-1/MAFbx, MURF-1, and the proteasomal assembly protein 26S proteasome non-ATPase regulatory subunit 11 (PSMD11) (Table 1). Relative expression (as fold change) of target genes were calculated after normalization to Beta-Actin using the 2^-ΔΔCt^ method.

**Table 1:** Gene primers for quantitative RT-PCR.

Gene	Genebank #	Primer Pairs	Tm/Product
**FOXO1**	NM_019739	Forward: 5’—CTACGAGTGGATGGTGAAGAGC Reverse: 5’—CCAGTTCCTTCATTCTGCACTC	64.9/5552
**MAFbx**	NM_026346	Forward: 5'—CTTCTCGACTGCCATCCTGGAT Reverse: 5'—TCTTTTGGGCGATGCCACTCAG	68.7/6936
**Murf-1**	NM_001039048	Forward: 5'—TACCAAGCCTGTGGTCATCCTG Reverse: 5'—ACGGAAACGACCTCCAGACATG	68.2/1886
**PSMD11**	XM_011249223	Forward: 5'—CAGCAGAGGAGAAGGACTGGAA	67/2693

### Body and soleus muscle mass

Body mass discriminated to 0.1g was measured on a calibrated analytic balance (Data Weighing Systems) at baseline (before survival surgery); and daily thereafter until necropsy (1-, 2-, or 4-weeks post-surgery). Soleus muscle tissue was harvested at necropsy as described above. Tissue wet weight was measured on a calibrated microbalance (Data Weighing Systems) to 0.01 mg, before being snap frozen using liquid nitrogen and stored at -80°C until the time of assay.

### Behavioral and motor function analysis

#### Open field and BMS test

The open field test was performed on experimental mice in an odor-free, non-transparent square arena as previously described. The arena was divided into three zones (wall, inter and center) and mouse behavior was recorded over a 5 min period using a high resolution, video camera. Total number of lines crossed, time spent in each zone and stereotypical behaviors such as grooming and rearing were analyzed and expressed as number of events. Mice that did not enter all three zones or cross a minimum of 50 lines during the 5 min trial were excluded. Scoring of locomotor hind-limb performance in the open field was performed with the BMS, [56] a 0 to 9 rating system based on a modification of the BBB and specifically designed for the mouse. Under blind conditions, a team of two investigators evaluates the mice over a 5-min time period 1 day after SCI and bi-weekly thereafter.

#### Rotarod time trial test

Motor coordination and balance were tested on the accelerating rotarod cylinder (Rotamex 4/8, Columbus Instruments). The test consisted of 5-day pre-training (days 1 to 5) followed by testing days at 2- and 4-weeks Post-SCI. The cylinder rotated at increasing speed and constant acceleration (from 10 to 60 rpm over 10 min period). The total time spent on the rod prior to fall was recorded and non-walking behaviors, such as passive clinging to the rod, were manually corrected. Each trial consisted of the average of 4 sessions. After each session, the mice were transferred back to their cage and allowed to rest for 20 min to avoid exhaustion and minimize stress. Mice that could not maintain their balance on the rod for a minimum of 60 seconds during pre-training were excluded from analysis.

#### Grip strength test

All experimental mice underwent analysis of hind-limb peak force (muscle strength) using the grip-strength test. Hind-limb grip strength was assessed using a digital force gauge (Chatillon DFIS2, Ametek) which generates a measure of neuromuscular function as maximal muscle strength—with the unit of force measured being delivered in Newton’s. The test consisted of a baseline assessment prior to surgery, followed by testing days at 2- and 4-weeks Post-SCI. Force values were calculated average of 5-trials.

### Statistical analysis

Between group differences were analyzed using one-way analysis of variance (ANOVA), followed by Tukey post hoc comparison and reflect fold change (immunoblot and mRNA analysis) and absolute change (soleus muscle mass and all behavioral analysis) from sham-control animals. For body mass, between group and within group differences across time were analyzed using a mixed-model analysis of variance (ANOVA), followed by Tukey post hoc comparison (GraphPad, Prism) and reflect fold change from sham control animals. Data are expressed as mean ± SEM. A significance level of p≤0.05 was accepted as different from control. n = 6 for each group, and each sample was run in triplicate (immunoblots) or duplicate (mRNA). n = 8–10 for each group (behavioral testing).

## Results

### UA treatment attenuates the loss of IGF-1/mTOR signaling observed following SCI

**Fig 1:**
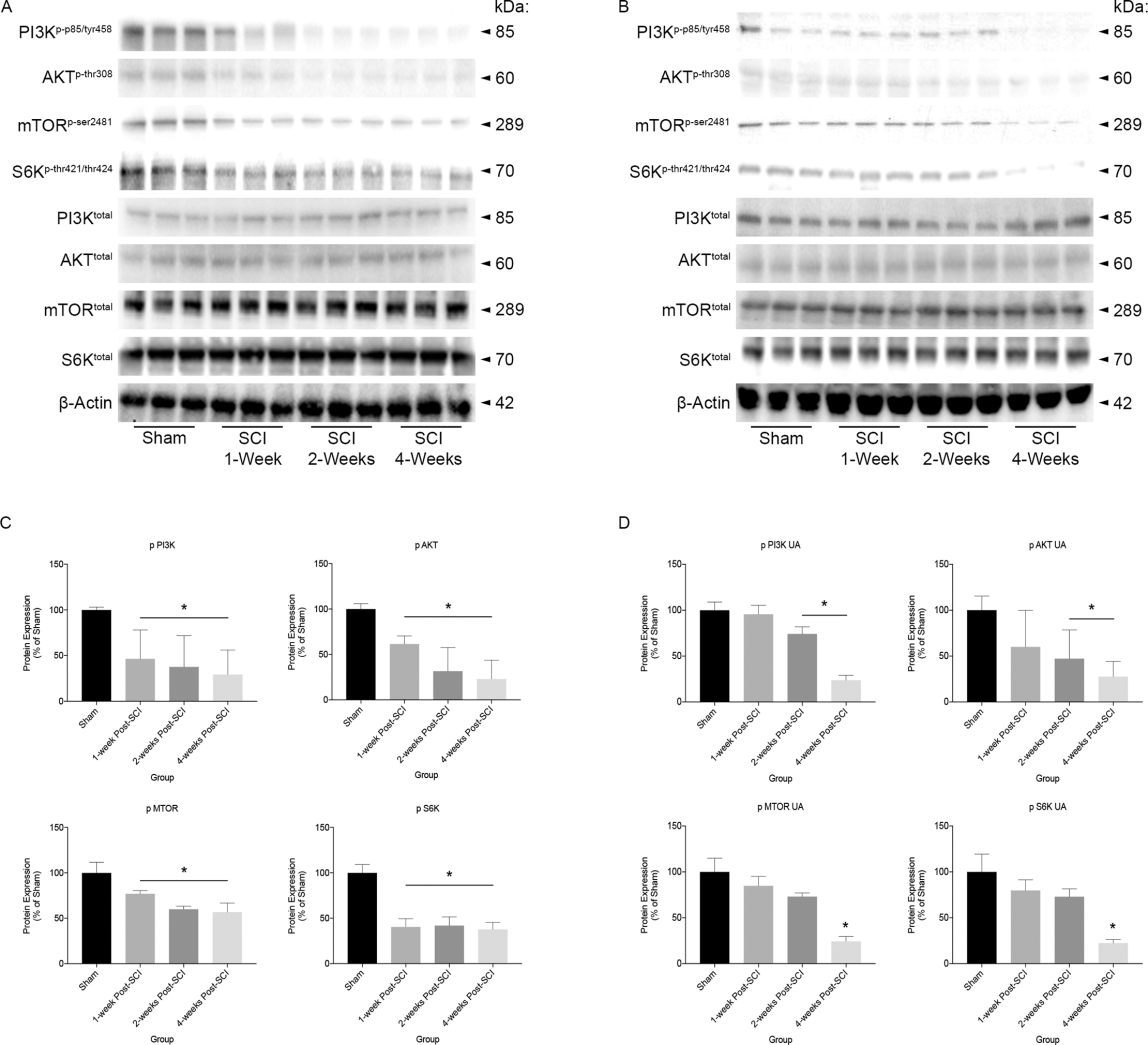
Phosphorylated protein expression of PI3K, AKT, mTOR, and S6K in soleus muscle from sham, SCI, and SCI-UA treated mice. At 1-, 2- and 4-weeks post-SCI, phosphorylated forms of PI3K, AKT, mTOR, and S6K were significantly decreased when compared to sham controls (**A,C**). In UA treated mice, there was no longer a significant difference at 1-week post-SCI compared to sham controls for all signaling intermediates. At 2- and 4-weeks post-SCI, there was a significant decrease in phosphorylated PI3K and AKT, and by 4-weeks only was there a significant decrease in phosphorylated mTOR and S6K compared to sham controls (**B,D**). *p < 0.05.

To define whether SCI alters activation of IGF-1/mTOR signaling cascades, and examine the effect of UA treatment, soleus muscle lysates from control (sham) and injured animals at various times after trauma were analyzed by immunoblotting procedures (Fig 1). We determined that SCI induced a significant decrease in the activated (phosphorylated) forms of PI3K, AKT, mTOR, and S6K at 1-, 2-, and 4-weeks post SCI (all P’s, 0.01), when compared to sham control (Fig 1A and 1C). However, in mice treated with UA, changes in expression of activated forms of all proteins examined were no longer significant at 1-week post-SCI compared to sham. By 2- and 4-weeks post-SCI phosphorylated PI3K (P’s < 0.01) and AKT (P’s < 0.05) were again significantly diminished, and only at 4-weeks post-SCI were phosphorylated mTOR and S6K significantly less compared to sham (both P’s < 0.01) (Fig 1B and 1D). Thus, SCI induces a significant decrease in activated mTOR and upstream signaling intermediates, and treatment with UA attenuates these signaling deficits acutely, delaying the effects of SCI on this IGF-1/mTOR pathway.

### UA treatment reduces FOXO1, MAFbx, MURF-1, and PSMD11 gene upregulation that occurs acutely following SCI

**Fig 2:**
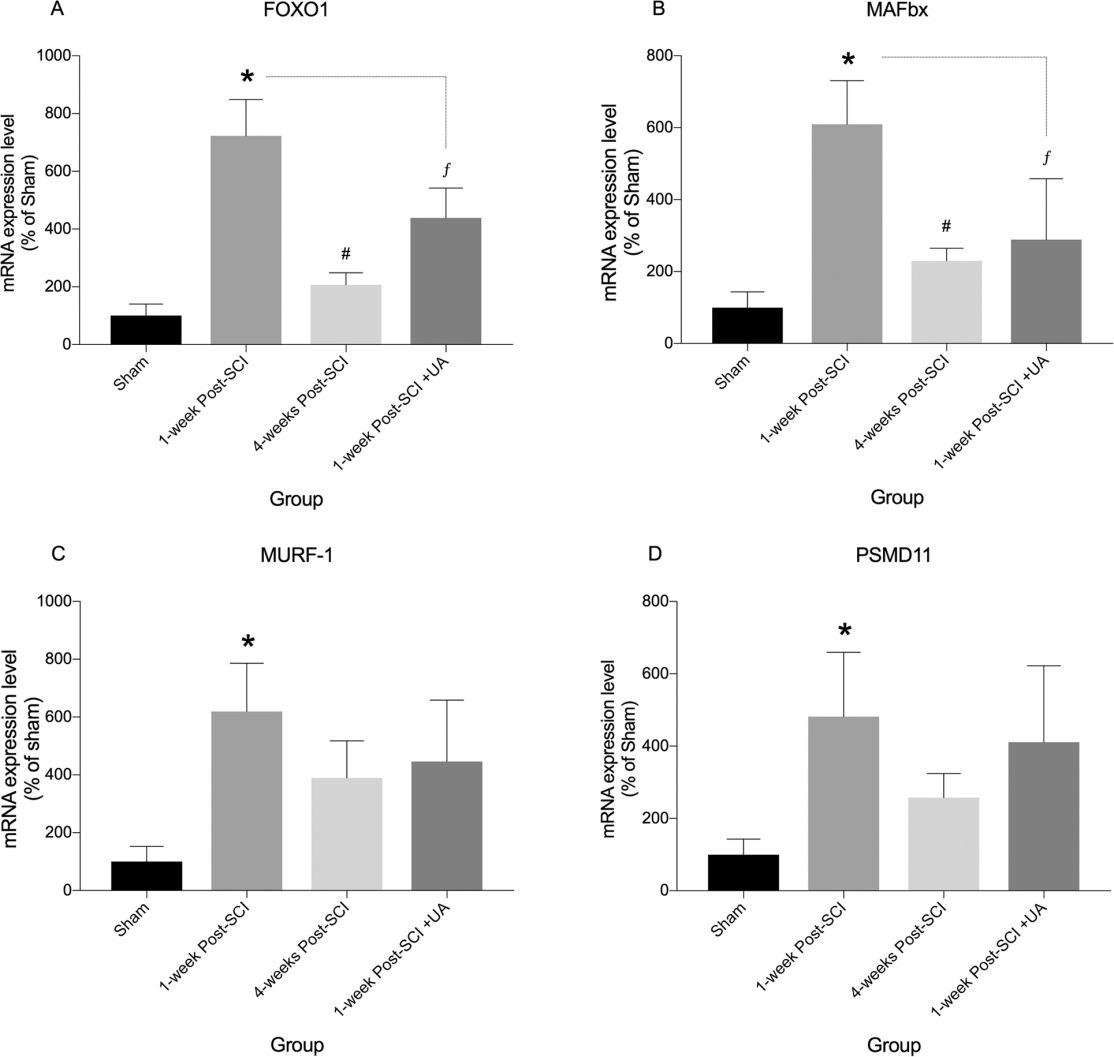
mRNA analysis of FOXO1, MAFbx, MURF-1, and PSMD11 in soleus muscle from Sham, SCI, and SCI-UA treated mice. mRNA expression of FOXO1, MAFbx, MURF-1 and PSMD11 were significantly greater at 1-week post-SCI (*) compared to sham control (**A-D**). By 4-weeks post-SCI, FOXO1 and MAFbx mRNA were significantly reduced compared to 1-week (**A,B** #) post-SCI and no longer significantly different from sham controls. At 4-weeks post-SCI Murf-1 and PSMD11 mRNA were observably but not significantly lower than 1-week post-SCI and no longer statistically different from sham controls (**C,D**). In UA treated mice, mRNA expression of FOXO1 and MAFbx were significantly less compared to untreated SCI mice at 1-week post-SCI (**A,B** *f*), and mRNA of MURF-1 and PSMD11 were observably but not statistically different at the same time-point compared to sham controls (**C,D**). *^,#, *f*^p < 0.05.

To investigate activation of catabolic processes in soleus muscle extracts from our experimental groups, we examined the gene expression levels of the transcription factor FOXO1, the muscle-restricted E3 ligases Atroginin/MAFbx and muscle ring finger-1 (MuRF-1), and the proteasomal protein PSMD11, using quantitative rt-PCR (Fig 2). Significant increases in mRNA expression were observed in all genes examined when compared to sham control, and peaked at 1-week post-SCI (FOXO1, MAFbx, P’s < 0.01; MuRF-1, PSMD11, P’s < 0.05) (Fig 2A–2D). By 4-weeks post-SCI, levels of FOXO1 and MAFbx mRNA were lower than levels observed at 1-week post-SCI and were commensurate with those of sham controls (Fig 2A and 2B). By 4-weeks post-SCI, MURF-1 and PSMD11 mRNA levels were no longer significantly different from those of sham animals, however the lower levels observed relative to 1-week post-SCI were not statistically significant (Fig 2C and 2D). Furthermore, in mice receiving UA treatment, at 1-week post-SCI, FOXO1 and MAFbx mRNA expression was significantly lower when compared to mice at 1-week post-SCI without treatment (i.e. peak expression; both P’s < 0.05) (Fig 2A and 2B). Non-significant decreases in MURF-1 and PSMD11 were also observed (Fig 2C and 2D). These results provide evidence that several catabolic genes associated with proteolysis are upregulated in soleus muscle acutely following SCI, and UA treatment is capable of thwarting these early effects.

### UA treatment preserves body and soleus muscle mass following SCI

**Fig 3:**
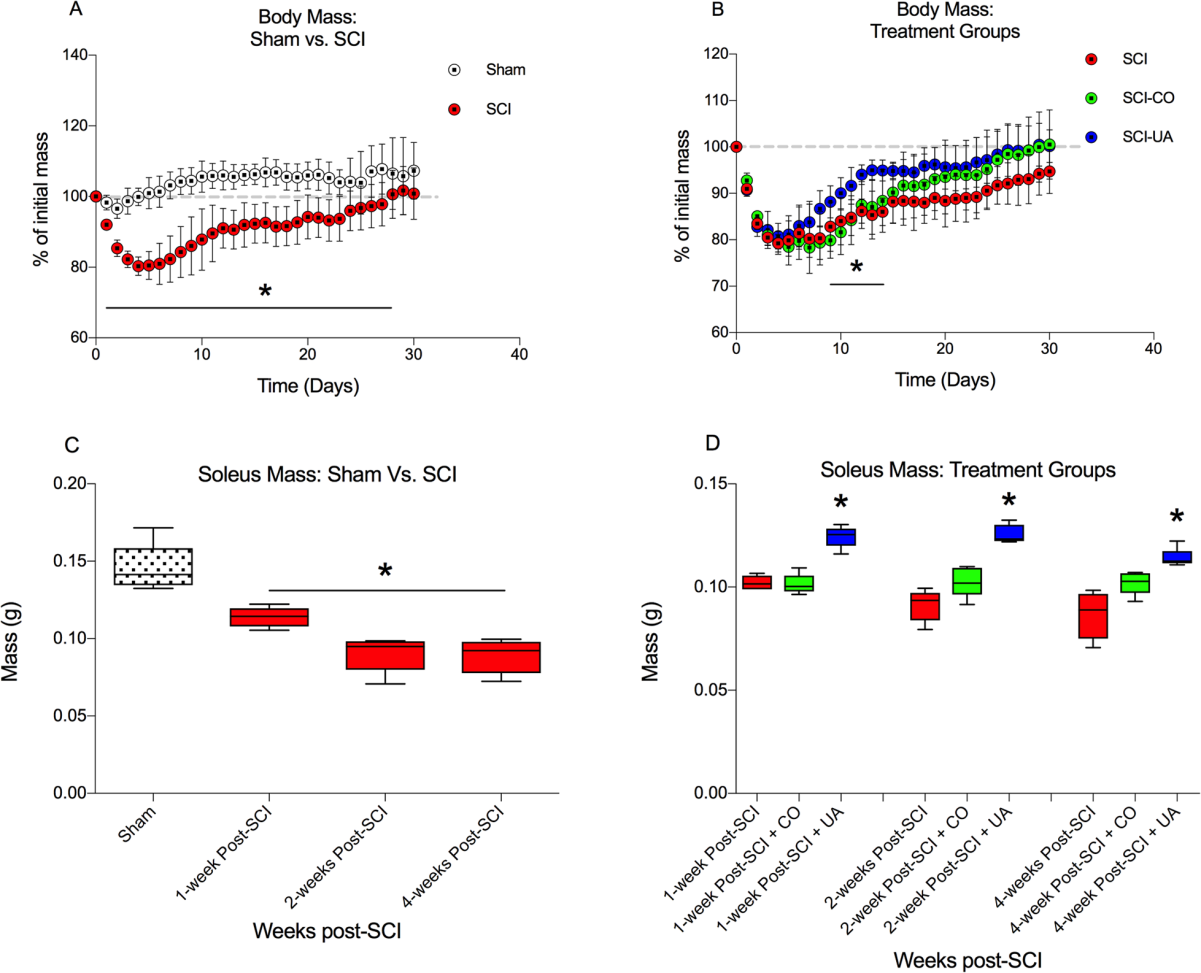
UA treatment preserves body and soleus muscle mass following SCI. **A.** Body mass was significantly reduced between 1–28 days post-SCI compared to sham controls, and progressively recovered to baseline by ~4-weeks post-SCI. **B.** UA treatment significantly increased body mass compared to SCI-CO (vehicle) and SCI alone between days 9–11 and 12–14, respectively. **C.** Soleus muscle mass was significantly reduced at 1-, 2-, and 4-weeks post-SCI compared to sham. **D.** UA treatment significantly increased Soleus muscle mass at all time-points compared to SCI-CO and SCI alone. *p < 0.05.

Body mass was measured at baseline (just prior to injury) and daily for 4-weeks post-SCI. Acutely post-SCI, there was a rapid and stark decrease in body mass which remained significant from 1-day post-SCI through 28 days (all P’s < 0.01 days 1–20; all P’s < 0.05 days 21–28) when compared to sham-control (Fig 3A). There was a progressive recovery of body mass in SCI mice which reached baseline level by ~4-weeks post-SCI, although still observably lower than sham by this time point. When compared to SCI and SCI-CO (vehicle control), in mice treated with UA (SCI-UA), there was an observable upward trend in body mass throughout the 4-week timeline, reaching significance at day 9–11 (all P’s < 0.05) compared to SCI-CO and day 12–14 (all P’s < 0.05) compared to SCI alone (Fig 3B). In addition, total soleus muscle mass (left and right) was significantly lower in tissue harvested at 1-, 2-, and 4-weeks post-SCI (all P’s < 0.01), when compared to sham control (Fig 3C). In SCI-UA, soleus muscle mass was significantly greater than SCI and SCI-CO control at all time-points post-SCI (1- and 2-weeks post-SCI, P’s <0.01; 4-weeks post-SCI, P’s <0.05) (Fig 3D). These data demonstrate that UA treatment may aide body mass maintenance, and importantly, preserve sub-lesional muscle mass after SCI.

### UA improves functional motor scores and measures of muscle coordination and sub-lesional muscle force production following SCI

**Fig 4:**
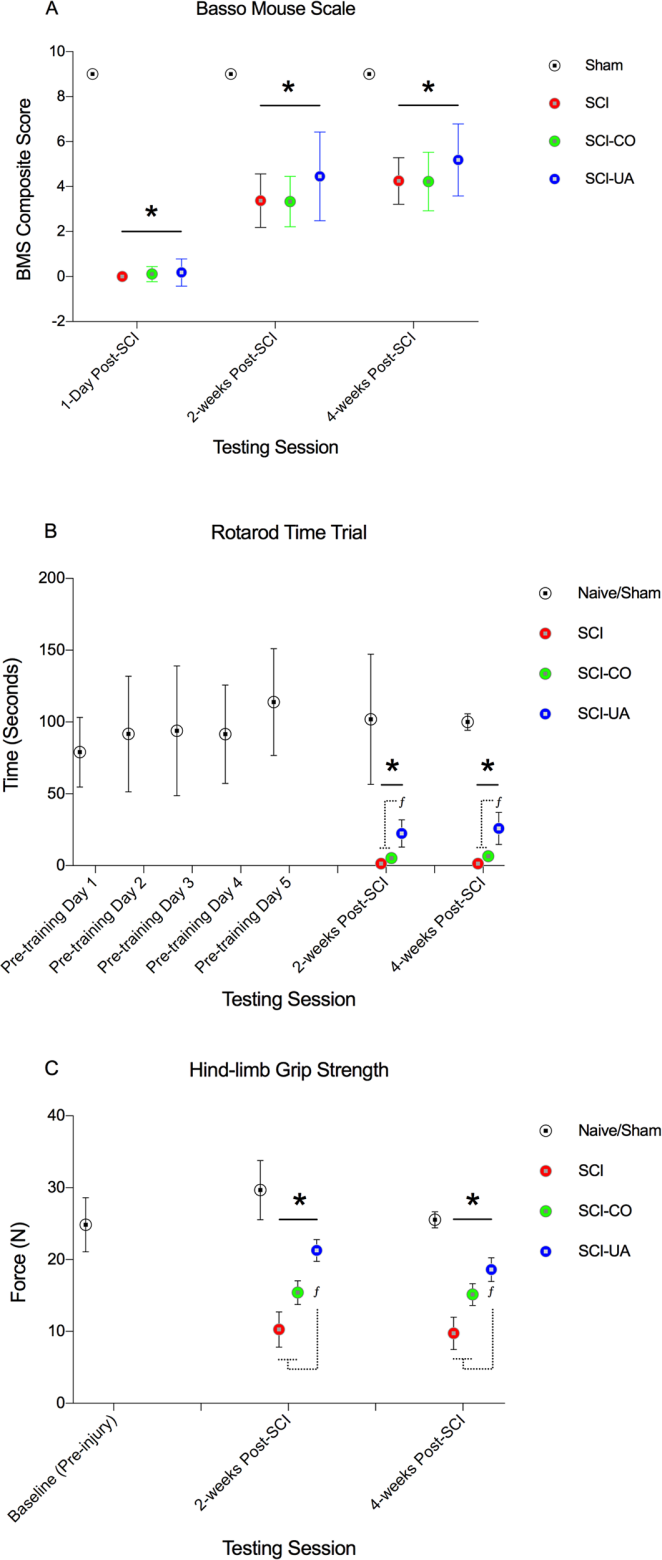
UA treatment improves sub-lesional locomotor outcomes and muscle force production following SCI. **A.** BMS composite score of locomotor function were significantly decreased in SCI, SCI-CO, and SCI-UA mice at 1-day, 2-, and 4-weeks post-SCI compared to sham controls. At 2- and 4-weeks post-SCI, there was a progressive increase in all groups, and an observably greater increase in UA treated mice compared to SCI-CO and SCI alone, although not statistically significant. **B.** Rotarod test time was significantly reduced in SCI, SCI-CO, and SCI-UA mice at both 2-, and 4-weeks post-SCI compared to sham controls. UA significantly improved rotarod time at all time-points compared to SCI-CO and SCI alone. **C.** Hind-limb muscle force production (grip strength) was significantly reduced in SCI, SCI-CO, and SCI-UA at 2- and 4-weeks post-SCI compared to sham controls. UA significantly improved grip strength at all time-points compared to SCI-CO and SCI alone. *^, *f*^p < 0.05.

To confirm the effects of SCI and UA treatment on sub-lesional motor function, we performed the BMS open field locomotor test, rotarod time trial, and hind-limb grip strength test (Fig 4). At 1-day post-SCI, significant deficits in BMS composite scores were observed in SCI, SCI-CO, and SCI-UA mice compared to sham controls (all P’s < 0.01) (Fig 4A). By 2- and 4-weeks post-SCI, BMS scores across all experimental groups remained significantly lower than sham controls, however, there was an observable yet statistically non-significant increase in BMS sores in SCI-UA compared to SCI-CO and SCI animals alone. Additionally, at 2- and 4-weeks post-SCI, all experimental groups showed significant deficits in Rotarod time trials when compared to sham controls (all P’s < 0.01) (Fig 4B). Moreover, SCI-UA mice were significantly greater compared to SCI-CO and SCI alone at the same time points post-SCI (both P’s < 0.05). Similarly, grip strength at 2- and 4-weeks post-SCI were significantly diminished in all experimental groups compared to sham controls (2-weeks post-SCI, P < 0.05; 4-weeks post-SCI, P <0.01) (Fig 4C), and SCI-UA mice exhibited significantly greater grip strength compared to SCI-CO and SCI alone at the same time points post-SCI (both P’s < 0.05). These results demonstrate that UA improves hind-limb function, motor coordination and grip strength after SCI.

## Discussion

In this study, we show that the natural compound UA improves anabolic IGF-1/mTOR signaling cascades in soleus muscle after experimental SCI. We also show that SCI-induced upregulation of several proteolytic genes, are reduced following treatment with UA. In addition, these genetic and biochemical effects of UA improve physical measures including: body and soleus muscle mass, and measures of muscle function, motor coordination and strength. These findings suggest that UA is a potential therapeutic adjunctive for physical deconditioning and motor deficits observed after SCI.

It is well established that the principal consequences of SCI on sub-lesional muscle include muscle atrophy [13, 22] and a slow and progressive transformation of fiber type resulting in a predominance of the glycolytic (type II) isoform. [8, 12, 40–44] These changes in the muscle promote two major outcomes: functional deficits of the paralyzed muscle due to the neurological insult and the development of metabolic dysfunction and cardiometabolic syndrome (CMS) risk factors. With respect to functional decline, previous reports using tests of maximum voluntary contraction [58] and isometric force production [59–61] have characterized atrophic muscle as having low contractile force. [62] Moreover, this muscle weakness is associated with rapid muscle fatigability, in both humans [12, 40–42] and animal models [43] [Reviewed in [63]], due to the predominantly glycolytic phenotype. Importantly, these pathological adaptations of the muscle have limited the “functional” recovery intended with leading rehabilitation strategies like extrinsically stimulated contractions using functional electrical stimulation (FES). Although select studies of long-term FES (6–12 months) have reported on atrophy reversal in acute SCI, [64] and the reversal of fiber type transformation in chronic SCI [65], functional improvements, albeit marginal, have only been observed with long-term FES (up to 16 months) in incomplete SCI. [66, 67] As such, the ability to provide a supplementary countermeasure to these pathological hallmarks on sub-lesional muscle may augment rehabilitation measures using FES and similar intervention paradigms.

SCI results in decentralization of neural connections and loss of contractile activity in the muscle. Although neuromuscular connectivity is maintained, there is impaired trophic support to the muscle, in which reduced neural activity contributes to disuse atrophy and progressive wasting similarly described in various contusion models, [68, 69] spinal isolation [70], denervation [71, 72], and human SCI. [58] The decrease in muscle work diminishes the tissue specific expression of IGF-1 and concomitant autocrine/paracrine mechanisms [73, 74] involving mTOR signaling cascades. [74, 75] Importantly, previous studies indicate downregulation of mTOR and signaling components (including S6K1) in models of chronic SCI. [72, 76] Relevant and opportune literature report that UA enhances IGF-1 and insulin mediated AKT [52] and S6K [53] signaling, two key intermediates in IGF-1/mTOR signaling. This effect of UA on IGF-1/mTOR signaling can independently inhibit atrophy and induce hypertrophy [53], as well as enhance muscle strength. [54] Here, we present data that builds off these previously reported findings and provide further evidence to support the ability of UA treatment to improve activation profiles of PI3K, AKT, mTOR, and S6K. Moreover, we also show that the effect of UA treatment on IGF-1/mTOR signaling translates to measurable physical and functional outcomes like sub-lesional muscle mass and grip strength, respectively, which are significantly greater when compared to untreated injured mice. These results suggest that IGF-1/mTOR signaling has a role in atrophy related pathological processes in SCI, and UA treatment has a protective effect via IGF-1/mTOR signaling that improves muscle related outcomes following SCI. It is important to note that UA may augment divergent pathways involved in hypertrophy. Glycogen synthase kinase 3 beta (GSK3β) is a substrate of AKT and represents an alternate muscle hypertrophy signaling pathway. GSK3β phosphorylation via AKT [77, 78] inactivates its enzymatic activity, which in turn releases 4E-binding protein 1 (4E-BP1) ultimately resulting in translation initiation and protein synthesis. [79] Recent reports also indicate G-protein-coupled receptor-mediated hypertrophy signaling [80] independent of IFG-1/ mTOR intermediaries (namely AKT). [81, 82] In particular, Gαi2 pathway has been shown to induce hypertrophy via PKC by negative regulation of GSK3β and activation of S6K1, respectively. This highlights the complexity of hypertrophy signaling cascades in muscle and the need to further examine both individual pathways and signaling integration to clarify the contribution of each pathway under atrophic conditions such as SCI, and better define the effects of treatment strategies such as UA.

Additionally, impaired mTOR signaling results in FOXO1 translocation to the nucleus where it activates catabolic genes involved in proteolysis and cell death. [49, 83–86] This represents a direct link to atrophic pathways involving catabolism and a point of divergence between hypertrophy and atrophy signals. In animal models of SCI and in acute SCI in humans [72, 76, 87, 88], mRNA expression of FOXO1 is altered, and two muscle-restricted E3 ligases, MAFbx and MuRF-1 –responsible for ubiquitin-mediated protein degradation in skeletal muscle [50]–are also altered, suggesting the ubiquitin-proteasome system (UPS) contributes to skeletal muscle proteolysis in SCI. Here we report the marked increase in FOXO1 mRNA, the *atrogenes* MAFbx, MURF-1 and the proteasomal assembly protein PSMD11 mRNA following SCI, demonstrating the mobilization of UPS-mediated degradation in the muscle. However, as we previously described above regarding hypertrophy signals, the observed increase in these catabolic genes cannot solely be attributed to IGF1/mTOR-mediated signaling, as several pathways are involved in atrophy linked *atrogene* expression and proteolysis in the muscle. For example, pro-inflammatory cytokine (including TNFα and IL-1) signaling activates NF-κB and p38 MAP kinase pathways that induce significant muscle atrophy via MAFbx [89–91] and MURF-1 [92–95] ubiquitin ligases. In addition, there is a growing body of literature confirming the effect of the tumor growth factor β (TGF-β) superfamily peptide, myostatin, in muscle wasting diseases (recently reviewed in [96]). Myostatin is a potent negative regulator of muscle growth [97–102] signaling through serine/threonine type kinase receptors to activate SMAD2/3 transcription factors. Importantly, SMAD target genes include (but are not limited to) ubiquitin-proteasome and autophagy-lysosome systems [103–106], as well as the suppression of myogenic regulatory factors. [107, 108] In addition to SMAD-mediated signaling, myostatin is shown to function in canonical protein synthesis and degradation signaling pathways including mTOR and FOXO1 [109, 110], again demonstrating the complexity and interplay between muscle hypertrophy and atrophy signaling pathways.

Importantly, myopenia occurring after SCI is associated with both substantial loss of sub-lesional muscle mass [13, 16] and fatty infiltration of muscle [16, 111], which together contribute to an obese somatotype [112], reduced whole-body metabolism [113, 114], and increased risk for cardiometabolic disease. [112] Previous rodent studies of SCI contusion and transection have confirmed changes in sub-lesional muscle size and phenotype, [43, 115–117] however, these changes have not been evaluated with respect to accompanying consequences. Beyond its notable trophic effects on skeletal muscle, UA reportedly promotes positive outcomes in models of diabetes and hyperlipidemia [118, 119], making it an attractive treatment strategy for attenuating secondary health complications associated with cardiometabolic disorders in SCI. Although we did not directly address these secondary complications, our data provide evidence that supports future studies to more comprehensively explore consequences of muscle pathology on global metabolism after SCI, and whether UA can mitigate these effects. For example, our current studies using experimental SCI examine phenotypic changes in muscle fiber type, whole body metabolism, serum analysis related to metabolic risk factors, and the relationship between these variables. One limitation here is that we administered UA dissolved in CO, consistent with literature describing UA treatment with atrophic stress. [53, 55] However, CO has a standardized composition which represents a considerable energy and fat source, evident when comparing the body weight curves and soleus weight for SCI, SCI/UA, and SCI/CO treatment groups. The CO alone as an energy substrate may augment body weight and potentially contribute to the lipid infiltration in sub-lesional muscle. Consequently, this obfuscated our examination of outcomes specific to metabolic dysfunction. Future studies will adopt oral or dietary UA administration [120–123] and chronic SCI survival to better address these questions.

## Conclusion

Currently there is a lack of effective rehabilitative therapies following SCI. Moreover, a paucity of literature has explored therapies directed at exploiting pathways that contribute to sarcopenia in SCI. Given that UA has shown potential in other paradigms of muscle atrophy, our findings provide evidence that supports the potential for UA to attenuate muscle-specific pathological consequences of SCI. Further research will help elucidate the potential effect of UA as a viable treatment strategy that may improve muscle health and function following SCI.

## Supporting information

S1 Table Chemiluminescence detection of target proteins in soleus muscle tissue. Shown are normalized relative intensities corresponding to chemiluminescence protein detection from immunoblots targeting the phosphorylated forms of PI3K, AKT, mTOR, and S6K. Groups include sham vs. SCI (1-, 2-, and 4-weeks), and SCI at the same timepoints with UA treatment.
(XLSX)

S2 Table Quantitative PCR CT Values for catabolic genes in soleus muscle. Shown are CT values for target genes FOXO1, MAFbx, MURF-1, and PSMD11 normalized to Actin (2^-ΔΔCt^).
(XLSX)

S3 Table Body mass curves and soleus muscle mass. A complete list of body mass at baseline and daily for 30 days post-SCI. Groups include sham, SCI, SCI with CO, and SCI with UA. Soleus muscle tissue was harvested at time of necropsy at 1-, 2- and 4-weeks post-SCI and mass was recorded. Groups include sham vs. SCI (1-, 2-, and 4-weeks), and SCI at the same timepoints with UA treatment.
(XLSX)

S4 Table Behavioral and motor function scores. Shown are BMS composite scores for hindlimb locomotor function, rotarod time for motor coordination and balance, and hindlimb grip strength. Values include baseline/pretraining, 1-day, 2- and 4-weeks post-SCI for BMS, and baseline/pretraining, 2- and 4-weeks post-SCI for both rotarod time trial and grip strength.
(XLSX)
